# Experiences of coercion to sterilize and forced sterilization among women living with HIV in Latin America

**DOI:** 10.7448/IAS.18.1.19462

**Published:** 2015-03-24

**Authors:** Tamil Kendall, Claire Albert

**Affiliations:** Women and Health Initiative, Department of Global Health and Population, Harvard School of Public Health Boston, MA, USA

**Keywords:** sterilization, HIV, reproductive rights, Latin America, El Salvador, Honduras, Mexico, Nicaragua, stigma, discrimination

## Abstract

**Introduction:**

Forced and coerced sterilization is an internationally recognized human rights violation reported by women living with HIV (WLHIV) around the globe. Forced sterilization occurs when a person is sterilized without her knowledge or informed consent. Coerced sterilization occurs when misinformation, intimidation tactics, financial incentives or access to health services or employment are used to compel individuals to accept the procedure.

**Methods:**

Drawing on community-based research with 285 WLHIV from four Latin American countries (El Salvador, Honduras, Mexico and Nicaragua), we conduct thematic qualitative analysis of reports of how and when healthcare providers pressured women to sterilize and multivariate logistic regression to assess whether social and economic characteristics and fertility history were associated with pressure to sterilize.

**Results:**

A quarter (23%) of the participant WLHIV experienced pressure to sterilize post-diagnosis. WLHIV who had a pregnancy during which they (and their healthcare providers) knew their HIV diagnosis were almost six times more likely to experience coercive or forced sterilization than WLHIV who did not have a pregnancy with a known diagnosis (OR 5.66 CI 95% 2.35–13.58 *p*≤0.001). WLHIV reported that healthcare providers told them that living with HIV annulled their right to choose the number and spacing of their children and their contraceptive method, employed misinformation about the consequences of a subsequent pregnancy for women's and children's health, and denied medical services needed to prevent vertical (mother-to-child) HIV transmission to coerce women into accepting sterilization. Forced sterilization was practiced during caesarean delivery.

**Conclusions:**

The experiences of WLHIV indicate that HIV-related stigma and discrimination by healthcare providers is a primary driver of coercive and forced sterilization. WLHIV are particularly vulnerable when seeking maternal health services. Health worker training on HIV and reproductive rights, improving counselling on HIV and sexual and reproductive health for WLHIV, providing State mechanisms to investigate and sanction coercive and forced sterilization, and strengthening civil society to increase WLHIV's capacity to resist coercion to sterilize can contribute to preventing coercive and forced sterilization. Improved access to judicial and non-judicial mechanisms to procure justice for women who have experienced reproductive rights violations is also needed.

## Introduction

Forced sterilization occurs when a person is sterilized without her knowledge or informed consent [1]. Coerced sterilization occurs when misinformation, intimidation tactics, financial incentives or access to health services or employment are used to compel individuals to accept the procedure [1]. Throughout history, social exclusion and discrimination based on ethnicity, social class, disabilities and health status has led to targeting of particular groups of women for coercive and forced sterilization [2–4]. The United Nations bodies responsible for monitoring compliance with international human rights law have condemned coerced and forced sterilization as a violation of the right to health, bodily integrity, the right to freedom from violence, freedom from torture and inhuman and degrading treatment, freedom from discrimination, and women's right to decide the number and spacing of children [4].

Coercive and forced sterilization of women living with HIV (WLHIV) has been reported in Africa [5–7], Asia [8] and Latin America [9,10]. HIV-related discrimination experienced by WLHIV when seeking reproductive health services and negative attitudes towards the reproduction of WLHIV reported by healthcare providers in many countries suggest that HIV status may be a critical driver of coercive and forced sterilization of WLHIV [11]. However, in a landmark Namibian court case, despite recognizing that WLHIV had been sterilized without their informed consent, the judge concluded that there was insufficient evidence that the women's HIV status motivated the forced sterilizations [12].

To assess whether characteristics other than HIV status may contribute to healthcare providers engaging in coercive and forced sterilization, we analyzed associations between social and economic characteristics and fertility history and experiencing pressure to sterilize from healthcare providers post-diagnosis among WLHIV from El Salvador, Honduras, Mexico and Nicaragua. We also consider women's accounts of how and when healthcare providers pressured them to undergo sterilization to elucidate the relationship between HIV-related discrimination and coercive and forced sterilization and the healthcare contexts that increase vulnerability. We conclude by making recommendations to protect WLHIV from this reproductive rights violation.

## Methods

### Data collection and sample

Information was collected using a questionnaire developed by feminist lawyers based on principles of international human rights law to document reproductive rights violations and subsequently adapted by 45 WLHIV and allies from the women's health movement. Participatory questionnaire development ensured appropriateness for the national legal frameworks, health systems, and sensitivity to cultural norms and the lived realities of WLHIV. The interviewer-applied questionnaire was administered in Spanish. The research instrument consisted primarily of multiple choice responses but incorporated open-text questions to elicit detail about WLHIV's experiences of reproductive rights violations; respondents could choose not to answer any question. In a section of the questionnaire that asked women if they had been pressured by healthcare providers to accept a contraceptive method because they were living with HIV, WLHIV were asked specifically “have you been forced or pressured to undergo sterilization” (yes or no), and to specify in an open-text response “how [they] were forced or pressured to accept a contraceptive method, including sterilization, and why.”

Community leaders were selected to participate in the development and application of the questionnaire because of their work with diverse groups of WLHIV, including transwomen and sex workers, and to represent different geographic areas of their countries. From July 2012 until February 2013, women leaders living with HIV and allies from the women's health movement invited WLHIV from their professional and social networks, for example, peers from HIV support groups, to complete the interviewer-applied questionnaire. Eligibility criteria for participation in the study was self-identifying as a woman or transwoman and having a confirmed HIV-positive diagnosis. In total, 337 WLHIV from 37 different political districts in El Salvador, Honduras, Mexico and Nicaragua completed the questionnaire. The total sample included 52 transwomen, none of whom reported experiencing pressure to sterilize, who were excluded from this analysis. The sample for this analysis included 285 WLHIV from four Mesoamerican countries.

All participants completed written or verbal informed consent before answering the questionnaire. Permission to analyze the de-identified data was granted to the authors by the Harvard School of Public Health Office of Regulatory Affairs and Research Compliance.

### Data analysis

Women's experiences of pressure to sterilize were analyzed using both qualitative and quantitative methods. The open-ended question asking WLHIV to describe their experiences of pressure to sterilize post-HIV diagnosis was analyzed thematically in Spanish, and selected representative quotes were translated into English. Multivariate logistic regression using maximum likelihood estimation was used to analyze the relationship between experiencing pressure to sterilize and women's social and economic characteristics and fertility history. To account for potential bias introduced by non-response, Rubin's multiple imputation (MI) method was used to create complete datasets by estimating and assigning missing values; analysis is then performed on each imputed dataset and results are pooled to provide efficient and statistically valid estimates [13]. While sufficiently efficient (90%+) estimates can be obtained through as few as three to five imputations [13,14], we followed Bodner's recommendation that the optimal number of imputations is roughly equivalent to the proportion of incomplete cases [15]. The original sample had 5% missing information with 45.6% incomplete cases so we created 46 imputed datasets during the MI process. To address possible clustering of responses because participants were recruited through peer-networks, we generated cluster-robust standard errors at the interviewer level, using Stata's clustered sandwich estimator.

The following variables were included as predictors in the multivariate model: ethnicity (Indigenous or of African descent), education (primary or less vs. secondary or more), marital status (married/cohabitating vs. single, divorced, separated or widowed), number of living children (none, one, or two or more), housing status (owners, borrowers, renters, or homeless), wealth indicators (internet at home, cement floor, using firewood for cooking), engaging in sex work and pregnancy with a known HIV-positive diagnosis. Women were defined as having a pregnancy with a known HIV-positive diagnosis if they reported 1) being diagnosed with HIV during pregnancy or childbirth or 2) becoming pregnant after they knew that they were living with HIV.

## Results

Of the 285 WLHIV included in this analysis, 56 were from El Salvador, 87 from Honduras, 82 from Mexico, and 60 from Nicaragua. Table 1 describes the social and economic characteristics and fertility history of the participants. Thirteen percent of women self-identified as Indigenous and 7% as being of African descent. The mean age of the women at the time of the interview was 37 years. The sample was divided almost evenly between women living with a stable partner (44%) and those who were not (56%). The mean number of living children was 2; 14% of the women had no living children, 15% had one living child, and 71% had two or more children. More than a third of the women (37%) had had a pregnancy with a known HIV diagnosis. Level of formal education and selected measures of wealth varied considerably. Experiencing pressure to sterilize was relatively common. In total, almost a quarter (23%) said they had been pressured to sterilize post-diagnosis: 17% in Nicaragua, 22% in Honduras, 23% in El Salvador and 28% in Mexico.

**Table 1:** Social and economic characteristics and fertility history of women living with HIV

	No.	%		No.	%
**Social characteristics**					
Age			Education		
≤24	23	8	None	19	7
25–34	100	36	Primary	137	49
35–44	89	32	Secondary	74	26
45+	67	24	More than Secondary	50	18
Married/cohabitating			Sex worker		
No	158	56	No	258	92
Yes	122	44	Yes	24	8
Indigenous			African descent		
No	240	87	No	252	93
Yes	37	13	Yes	20	7
**Economic characteristics**					
Housing status			Home Internet		
Owners	145	51	No	231	82
Borrowers	73	26	Yes	51	18
Renters	59	21			
Homeless	7	2			
Firewood for cooking			Cement floor		
No	232	82	No	132	47
Yes	50	18	Yes	150	53
**Fertility history**					
Number of living children			Pregnancy with a known HIV diagnosis		
None	40	14	No	133	63
One	43	15	Yes	77	37
Two or more	198	71			

### HIV-related discrimination and pressure to sterilize

In all four countries women were told by healthcare providers that their HIV status meant that they could not have more children and that they had to accept sterilization. An illustrative report was made by a woman from El Salvador who said “They forced me [into sterilization] because they told me that a person with HIV couldn't have more children” (31 years old, married, one child, El Salvador). Women were also told by healthcare providers that they could not exercise choice about their contraceptive method, for example, using condoms to prevent both pregnancy and HIV transmission, but rather had to accept sterilization because of their HIV status. For instance a woman from Mexico reported that healthcare providers “told me that because of my HIV problem, I couldn't refuse [sterilization]” (40 years old, divorced, two children, Mexico).

### Coercion to sterilize through misinformation

Despite the fact that at Latin American facilities rates of vertical (mother-to-child) HIV transmission have been reduced below 2% [16] and that antiretroviral therapy to prevent vertical transmission is available in El Salvador (since 2002) [17], Honduras (since 2004) [18], Mexico (since 1998) [19] and Nicaragua (since 2000) [20], sterilization was presented to WLHIV as an intervention to prevent vertical HIV transmission. A typical experience was described by a Nicaraguan woman who said that healthcare providers “told me that because I have this disease, my children could be born with HIV and that the best would be for me to be sterilized” (36 years old, separated, four children, Nicaragua). Women also reported being described as vectors of HIV disease by healthcare providers to pressure them to accept sterilization. For instance, women were told by healthcare providers to “get sterilized so as not to have more infected children in the world” (17 years old, single, one child, Mexico), or informed by healthcare providers that they would be sterilized “so that children wouldn't keep on being born with HIV” (21 years old, married, one child, Nicaragua).

WLHIV also reported that healthcare providers frightened them into accepting sterilization by stating that if they failed to do so, they or their children were likely to die. For example, a young woman from Honduras who explicitly stated that she “wanted to have another child” reported that “to sterilize me, they told me that if I got pregnant again, I could die” (24 years old, cohabitating, three children, Honduras). Threats to children's health were also used to coerce women into sterilization, as in this illustrative quote from a Nicaraguan woman who stated:

In the hospital, they told me that I couldn't have more children because I have HIV and that they had to sterilize me to stop me from giving birth again, and if I didn't do it, I had to protect myself [use a condom] because if I didn't, my child would die. (36 years old, single, two children, Nicaragua)

WLHIV consented to unwanted sterilization because they did not have accurate or adequate information about HIV or how to exercise their sexual and reproductive rights while living with HIV:

Maybe they didn't force me, but by not giving me any options and information, I was obliged to be sterilized. If they had given me the correct information, I wouldn't have accepted sterilization. (33 years old, cohabitating, two children, Nicaragua)

Asymmetries in access to information and power between WLHIV and healthcare providers made it difficult for women to resist pressure to sterilize. A Honduran woman explained,

They make a misleading proposal to the patients, and some of us accept because of the deception, and others because of their situation [HIV] or simply because they are afraid of retaliation from the system. (29 years old, married, two children, Honduras)

### Sterilization as a condition to receive interventions to prevent vertical HIV transmission

Sterilization was also presented to WLHIV implicitly or explicitly as a condition for receiving medical services and benefits, including caesarean delivery and breast-milk substitution used to prevent vertical HIV transmission. Frequently, women were pressured into signing consent for sterilization just prior to entering the operating theatre for a caesarean section. Women's vulnerability in this context was expressed clearly by a Mexican woman who said, “I was in labour, and what I wanted was to receive care. Dr. [X] really pressured me to accept sterilization, saying, ‘What kind of life are you going to give to your child?’” (27 years old, cohabitating, two children, Mexico). In other cases, sterilization was made an explicit condition for receiving medical treatment, as for this woman from El Salvador: “The nurses forced me to sign [consent for sterilization]. They asked me more than three times and threatened not to perform the caesarean. Because of the pressure, I had no option but to sign” (19 years old, separated, one child, El Salvador). Women also reported being told by healthcare providers that they would not receive economic support, such as formula, unless they were sterilized: “They forced me to accept sterilization by telling me that if I didn't, they wouldn't help me with milk for my children” (35 years old, married, three children, El Salvador).

### Forced sterilization

Finally, WLHIV reported being sterilized without their knowledge or consent. This abuse was identified in all four countries and in every instance occurred when women were under the effects of anaesthesia, administered to perform a caesarean section or another type of surgery. In one case, healthcare providers fabricated a fraudulent consent by making a mark of the WLHIV's thumbprint as a substitute for her signature while she was under the effects of anaesthesia:

During the caesarean, and under the effects of anaesthesia, they forced her to be sterilized so she couldn't have more children. She didn't sign any authorization, rather when she was recovering from the anaesthesia she saw that her thumb was stained with ink. (27 years old, cohabitating, two children, Mexico)

Other women stated that they had signed medical consent for caesarean section or other types of surgery, but reported that they had not knowingly consented to sterilization.

### Associations between social and economic characteristics, fertility history and experiencing pressure to sterilize

In the multivariate analysis, the statistically significant predictors of being more likely to experience pressure to sterilize were having had a pregnancy with a known HIV-positive diagnosis and being in the youngest age group (Table 2). Women who had a pregnancy during which their HIV diagnosis was known had almost six times the odds of reporting pressure to sterilize than WLHIV who did not have a pregnancy during which their HIV status was known. Women in older age groups (25–34 years of age and 45 or older) were less likely to report experiencing pressure to sterilize than women 24 years of age or younger.

**Table 2:** Multivariate analysis of relationships between fertility history, social and economic characteristics of women living with HIV and experience of pressure to sterilize: multiple imputation model

Observations=285	Imputations=46	
	
Characteristics	Odds ratio (Confidence Interval)	*p*
**Pregnancy after HIV diagnosis**	5.66*** (2.35–13.58)	≤0.001
**Living children**		
None	Referent	
One	1.59 (0.27–9.38)	0.606
Two or more	3.38 (0.56–20.59)	0.185
**Age at interview**		
≤24	Referent	
25–34	0.18* (0.04–0.91)	0.037
35–44	0.32 (0.07–1.58)	0.163
45+	0.07** (0.01–0.53)	0.010
**Education**		
Primary or less	Referent	
Secondary or more	1.68 (0.69–4.08)	0.255
**Married/cohabitating**	1.21 (0.56–2.64)	0.628
**Sex worker**	0.19 (0.02–1.94)	0.161
**Indigenous**	0.95 (0.31–2.87)	0.926
**African descent**	0.54 (0.10–2.87)	0.468
**Housing status**		
Owners	Referent	
Borrowers	0.52 (0.21–1.28)	0.157
Renters	2.06 (0.72–5.83)	0.176
Homeless	1.32 (0.20–8.65)	0.769
**Home Internet**	2.31 (0.81–6.60)	0.118
**Firewood for cooking**	0.81 (0.23–2.83)	0.745
**Cement flooring**	0.77 (0.37–1.60)	0.485

* *p*≤0.05

** *p*≤0.01

*** *p*≤0.001.

### Pregnancy while living with HIV and vulnerability to coercive and forced sterilization

Women who were diagnosed during prenatal care reported being vulnerable to coercion because of their lack of knowledge and limited time to assimilate the HIV diagnosis, while women who became pregnant after knowing that they were living with HIV were vulnerable because of the stigmatizing normative assumption that they were not “supposed” to get pregnant. To illustrate, a Mexican woman who learned of her HIV diagnosis during antenatal care explained that she was unable to refute the misleading information that the attending physician used to pressure her into sterilization after her caesarean delivery:

I was 23 years old, and the doctor who performed [the caesarean section] asked me if I was going to use a contraceptive method. I told him that condoms were good. He asked how I could dare to say that knowing I had HIV, and asked me who was going to take care of them [my children] when I died, and a whole bunch of other things. They practically forced me. And on top of that, I didn't know anything about HIV, and so, I had no choice. (26 years old, single, two children, Mexico)

WLHIV reported that healthcare providers viewed becoming pregnant after the diagnosis as a transgression and presented sterilization as the consequence. For instance, a Nicaraguan woman said she was informed by her physician that she would be sterilized because she had “disobeyed” his instruction not to become pregnant:

The doctor told me that he was going to sterilize me because of my problem [HIV]. And when I got pregnant, he told me that he had warned me not to have another child because of this problem—so he said, “we're going to sterilize you”. (29 years old, cohabitating, four children, Nicaragua)

## Discussion

Qualitative and quantitative analyses of reports by WLHIV from 37 different political districts in four Mesoamerican countries indicate that HIV-positive status is a central motivation for healthcare providers to pressure women to undergo surgical sterilization. Women's reports of how healthcare providers tried, and often succeeded, to coerce them into sterilization provides evidence for discrimination based on HIV status, as well as the misinformation and abuse of power by healthcare providers that characterizes coercive sterilization. WLHIV reported being told they could not have children because they were living with HIV, that sterilization was their sole contraceptive option or means of preventing vertical HIV transmission, and threatened with the spectre of maternal and infant mortality. The discriminatory arguments made by healthcare providers in El Salvador, Honduras, Mexico and Nicaragua to pressure WLHIV into sterilization are similar to those reported in South Africa [6], Namibia [5], Chile [9] and five Asian countries [8]. Our study builds upon prior reports of coercive and forced sterilization by WLHIV, and makes an additional contribution to the literature by exploring the relationships between coercive and forced sterilization and WLHIV's social and economic characteristics and fertility history. In particular, to our knowledge, the association between WLHIV having a pregnancy with a known HIV diagnosis and experiencing pressure to sterilize has not been previously analyzed.

The importance of promoting and protecting the reproductive rights of WLHIV in maternity care is brought into sharp focus by our finding that women who had a pregnancy during which they (and their healthcare providers) knew they were living with HIV were almost six times more likely to experience pressure to sterilize than WLHIV who did not have a pregnancy with a known HIV diagnosis. This finding is reinforced by WLHIV's descriptions of how pregnancy and seeking maternal health services increased their vulnerability to coercive and forced sterilization. Women diagnosed with HIV during pregnancy said they were susceptible to pressure because of their lack of knowledge about HIV and limited time to assimilate the HIV diagnosis, while women who had a pregnancy after learning their diagnosis were stigmatized and had sterilization presented by healthcare providers as a consequence of their “transgression.” WLHIV also reported that healthcare providers threatened to withhold labour and delivery services as a means of coercing them into sterilization. Finally, as documented in Africa [5,6] and other countries in Latin America [9,10] WLHIV who participated in this research reported that caesarean section and other abdominal surgeries were used by healthcare providers as opportunities to practice forced sterilization.

It is also notable that the multivariate analysis found that older WLHIV were less likely than women in the youngest age group to report experiencing pressure to sterilize. The World Health Organization recommends that even when young women explicitly request sterilization, healthcare providers should exercise caution in the provision of this permanent contraceptive method because young age is one of the strongest predictors of sterilization regret [21]. This finding provides additional evidence that HIV-related discrimination, rather than healthcare provider concerns about providing WLHIV with effective contraceptive methods appropriate to women's fertility desires and life circumstances, is contributing to WLHIV's experiences of coercive and forced sterilization.

The study has limitations and strengths. The convenience sample is not representative of WLHIV in the respective countries, which limits the generalizability of the findings. Another study limitation is the high occurrence of missing data (see Supplementary file). We attribute missing data to the application of questionnaires by community-based leaders rather than professional data collectors. Because the assumption that data is missing completely at random is unlikely to hold and an analysis of complete cases could be biased, we employed MI to predict missing data using observed covariates. Despite these limitations, the study has a number of strengths. The participant WLHIV came from more than 37 political districts in 4 countries, suggesting our findings have relevance beyond a single country or healthcare delivery site. Also, analysis of qualitative and quantitative data from the questionnaire allowed us to explore associations between WLHIV's social and economic characteristics and fertility history and occurrence of coercive and forced sterilization, as well providing insight into the arguments that healthcare providers used to coerce women and the contexts that increased women's vulnerability.

## Conclusions

The experiences of WLHIV from four Mesoamerican countries indicate that HIV status and HIV-related discrimination are key drivers of coercive and forced sterilization, and that protecting and promoting the reproductive rights of WLHIV seeking maternal health services should be a specific focus for action.

Based on our analysis, we recommend several actions to protect WLHIV from coercive and forced sterilization. First, we recommend pre-service and in-service training on HIV, sexual and reproductive rights and basic human rights principles, including non-discrimination and informed consent, for healthcare providers [22]. To promote respectful maternity care for all women and because of WLHIV's vulnerability to forced and coerced sterilization during labour and delivery, we recommend prioritizing training of healthcare professionals providing intrapartum care. Second, ensuring the option of vaginal rather than caesarean delivery is available to WLHIV when medically indicated can reduce opportunities for forced sterilization, as well as promote maternal health [23–25]. Third, to make informed decisions about reproduction, all WLHIV require comprehensive evidence-based education and counselling about the effectiveness of interventions to prevent vertical HIV transmission [16,26], access to a full range of contraceptive options [21], and information to support safer conception [27,28], as well as information about how to live a long and healthy life with HIV. This information should be an integral part of HIV counselling and a mandatory component of informed consent for sterilization. Finally, for States to fulfil their international human rights commitments to non-discrimination and the protection and promotion of the right to health, including sexual and reproductive rights, they must implement mechanisms to investigate cases of coercive and forced sterilization, sanction perpetrators and provide reparation to the women whose rights have been violated [22]. Strengthening civil society capacity, particularly of networks of WLHIV, to effectively use judicial and non-judicial mechanisms to protect rights and procure justice is one avenue to hold States and healthcare institutions accountable to their obligations to protect and promote the reproductive rights of WLHIV.

## Supplementary Material

Experiences of coercion to sterilize and forced sterilization among women living with HIV in Latin America

## References

[1] Open Society Foundation (2011). Against her will: forced and coerced sterilization of women worldwide.

[2] Miranda JJ, Yamin AE (2004). Reproductive health without rights in Peru. Lancet.

[3] Holt E (2012). Uzbekistan accused of forced sterilisation campaign. Lancet.

[4] Zampas C, Lamačková A (2011). Forced and coerced sterilization of women in Europe. Int J Gynaecol Obstet.

[5] Ahmed A, Roseman MJ, Gatsi-Mallet J (2012). “At the hospital there are no human rights”: reproductive and sexual rights violations of women living with HIV in Namibia.

[6] Strode A, Mthembu S, Essack Z (2012). “She made up a choice for me”: 22 HIV-positive women's experiences of involuntary sterilization in two South African provinces. Reprod Health Matters.

[7] Mallet J, Kalambi V (2008). Coerced and forced sterilization of HIV-positive women in Namibia. HIV AIDS Policy Law Rev.

[8] Women of the Asia Pacific Network of People Living with HIV (2012). Positive and pregnant: how dare you: APN+ [Internet]. http://www.apnplus.org/main/share/publication/APN+%20Reproductive%20and%20Maternal%20Health%20Report%20A4%2013%20April.pdf.

[9] Center for Reproductive Rights/Vivo Positivo (2010). Dignity denied: violation of the rights of HIV-positive women in Chilean health facilities.

[10] Kendall T (2009). Reproductive rights violations reported by Mexican women with HIV. Health Hum Rights.

[11] MacCarthy S, Rasanathan JJK, Ferguson L, Gruskin S (2012). The pregnancy decisions of HIV-positive women: the state of knowledge and way forward. Reprod Health Matters.

[12] (2012). Government of the Republic of Namibia v L.M. and Others, High Court of Namibia.

[13] Rubin DB (1987). Multiple imputation for nonresponse in surveys.

[14] Schafer JL (1997). Analysis of incomplete multivariate data.

[15] Bodner TE (2008). What improves with increased missing data imputations?. Struct Equ Modelling.

[16] Read JS, Cohen RA, Hance LF, Machado ES, Mussi-Pinhata MM, Ceriotto M (2012). Missed opportunities for prevention of mother-to-child transmission of HIV-1 in the NISDI Perinatal and LILAC cohorts. Int J Gynecol Obstet.

[17] World Health Organization (2005). El Salvador: summary country profile for HIV/AIDS treatment scale-up.

[18] World Health Organization (2005). Honduras: summary country profile for HIV/AIDS treatment scale-up.

[19] Saavedra-Lopez J, Bravo-Garcia E, Magis-Rodríguez C, Barrientos-Bárcenas H, Bertozzi-Kenefick S (2006). Panorama del VIH/SIDA en el 2006. SIDA: Aspectos de Salud Pública.

[20] World Health Organization (2005). Nicaragua: summary country profile for HIV/AIDS treatment scale up.

[21] World Health Organization (2010). Medical eligibility criteria for contraceptive use.

[22] African Commission on Human and Peoples’ Rights (2013). Resolution on involuntary sterilization and the protection of human rights in access to HIV services.

[23] Panel on Treatment of HIV-Infected Pregnant Women and Prevention of Perinatal Transmission (2014). Recommendations for use of antiretroviral drugs in pregnant HIV-1-infected women for maternal health and interventions to reduce perinatal HIV transmission in the United States [Internet]. http://aidsinfo.nih.gov/contentfiles/lvguidelines/PerinatalGL.pdf.

[24] Calvert C, Ronsmans C (2013). HIV and the risk of direct obstetric complications: a systematic review and meta-analysis. PLoS One.

[25] PAHO/UNICEF (2009). Guía clínica para la eliminación de la transmisión materno-infantil del VIH y de la sífilis congénita en América Latina y el Caribe.

[26] Townsend CL, Byrne L, Cortina-Borja M, Thorne C, de Ruiter A, Lyall H (2014). Earlier initiation of ART and further decline in mother-to-child HIV transmission rates, 2000–2011. AIDS.

[27] Matthews LT, Smit JA, Cu-Uvin S, Cohan D (2012). Antiretrovirals and safer conception for HIV-serodiscordant couples. Cur Opin HIV AIDS.

[28] Bekker L-G, Black V, Myer L, Rees H, Cooper D, Mall S (2011). Guideline on safer conception in fertile HIV-infected individuals and couples. South Afr J HIV Med.

