# Supplementary material for: Experiences of coercion to sterilize and forced sterilization among women living with HIV in Latin America
Source: J Int AIDS Soc. 2015 Mar 24;18(1):19462. doi: 10.7448/IAS.18.1.19462 (PMC4374084; doi:10.7448/IAS.18.1.19462)
Supplement: Experiences of coercion to sterilize and forced sterilization among women living with HIV in Latin America [file JIAS-18-19462-s001.pdf]

Additional File 1

Table 3: Social and economic characteristics and fertility history of women living with HIV (frequency of missing values)

Social Characteristics					
Age	#	%	***Education***	#	%
≤24	23	8.1	None	19	6.7
25-34	100	35.1	Primary	137	48.1
35-44	89	31.2	Secondary	74	26.0
45+	67	23.5	More than Secondary	50	17.5
Missing	6	21	Missing	5	1.8
Married/Cohabitating	#	%	***Sex Worker***	#	%
No	158	55.4	No	258	90.5
Yes	122	42.8	Yes	24	8.4
Missing	5	1.8	Missing	3	1.1
Indigenous	#	%	***African Descent***	#	%
No	240	84.2	No	252	88.4
Yes	37	13.0	Yes	20	7.0
Missing	8	2.8	Missing	13	4.6
Economic Characteristics					
Housing Status	#	%	***Home Internet***	#	%
Owners	145	50.9	No	231	81.1
Borrowers	73	25.6	Yes	51	17.9
Renters	59	20.7	Missing	3	1.1
Homeless	7	2.5			
Missing	1	0.4			
Firewood for Cooking	#	%	***Cement Floor***	#	%
No	232	81.4	No	132	46.3
Yes	50	17.5	Yes	150	52.6
Missing	3	1.1	Missing	3	1.1
Fertility History					
Number of living children	#	%	***Pregnancy with a known HIV diagnosis***	#	%
None	40	14.0	No	133	46.7
One	43	15.1	Yes	77	27.0
Two or More	198	69.5	Missing	75	26.3
Missing	4	1.4			

Table 3 provides the frequencies of missing data for the social and economic characteristics and fertility history of the participant women living with HIV.
